# Supplementary material for: Anisotropic magnetoresistance in spin–orbit semimetal SrIrO3
Source: Eur Phys J Plus. 2020 Aug 5;135(8):627. doi: 10.1140/epjp/s13360-020-00613-3 (PMC7411514; doi:10.1140/epjp/s13360-020-00613-3)
Supplement: Supplementary file 1 — Supplementary material 1 (pdf 628 KB) [file 13360_2020_613_MOESM1_ESM.pdf]

Supplementary information for: Anisotropic magnetoresistance in spin-orbit semimetal $SrIrO_3$

D. J. Groenendijk,* N. Manca, J. de Bruijckere, A. M. R. V. L. Monteiro, R. Gaudenzi, H. S. J. van der Zant, and A. D. Caviglia

Kavli Institute of Nanoscience, Delft University of Technology, Lorentzweg 1, 2628 CJ Delft, Netherlands

Dated: January 20, 2020

CONTENTS

I. Growth of SIO and STO thin films 2

II. Magnetoresistance of 5 and 30 u.c. SIO films 2

* d.j.groenendijk@tudelft.nl

I. GROWTH OF SIO AND STO THIN FILMS

Figure S1 shows the RHEED intensity during the growth of 5, 6, and 30 u.c. SIO films on TiO_2-terminated STO(001) substrates. The growth of SIO is followed by the growth of a 10 u.c. STO film to enable patterning of Hall bars and prevent degradation of the SIO. The clear intensity oscillations indicate that both the SIO and STO grow in layer-by-layer mode.

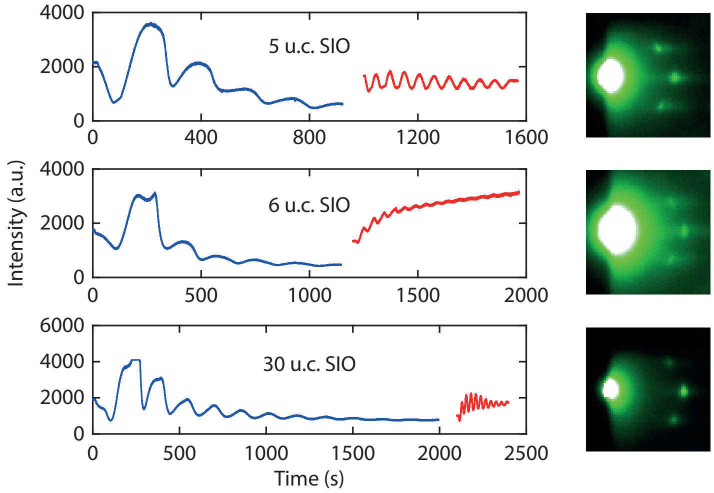

FIG. S1: **RHEED during the growth of SIO and STO.** From top to bottom: RHEED oscillations during the growth of 5 u.c., 6 u.c., and 30 u.c. SIO films, followed by 10 u.c. STO. For the 30 u.c. film, only 12 oscillations are shown. Right: RHEED pattern after the growth of SIO.

II. MAGNETORESISTANCE OF 5 AND 30 U.C. SIO FILMS

Magnetoresistance (MR) measurements of 30 and 5 u.c. SIO films are shown in Fig. S2. The magnitude of the MR of the 30 u.c. film increases with decreasing temperature (Fig. S2a). Below 4.2 K, the MR displays a cusp at low fields which can be attributed to quantum corrections.

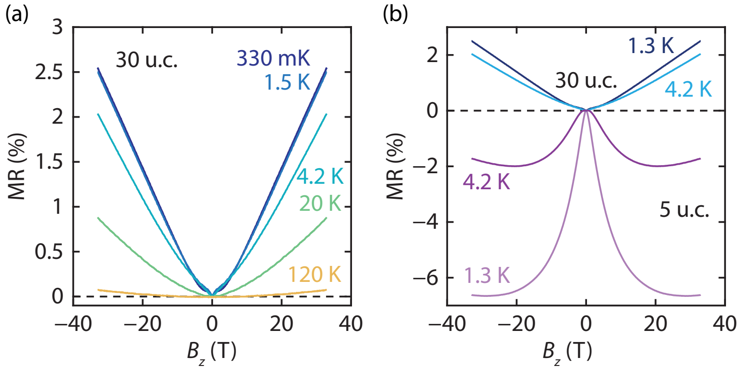

FIG. S2: **Temperature evolution of the MR of 30 and 5 u.c. SIO films.** (a) Temperature-dependent MR of the 30 u.c. SIO film. (b) MR of the 30 and 5 u.c. SIO films measured at 4.2 K and 1.3 K.

Figure S2 shows the MR of the 30 and 5 u.c. SIO films measured at 4.2 K and 1.3 K. The 5 u.c. film displays a large negative MR due to weak localization.

Figure S3a shows the MR of the 30 and 5 u.c. films measured at 1.3 K for B oriented parallel (red) and perpendicular (black) to the film normal.

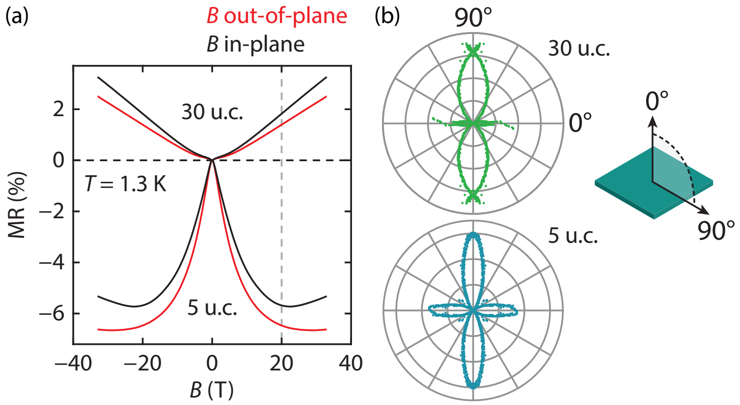

FIG. S3: **Angular dependence of the MR.** (a) MR of the 30 and 5 u.c. SIO films for $B \parallel n$ (red) and $B \perp n$ (black), where n is the film normal. (b) Polar plots of the MR. To the right, a schematic of the two orientations is shown.

For both films, the negative MR is larger when B is oriented in the film plane. To study the anisotropy in more detail, the MR is measured as a function of angle with an applied magnetic field of 20 T. The polar plots in Fig. S3b show two large and two small lobes, similar to the measurements on the 6 u.c. film shown in Fig. 2b (bottom) and 3a (bottom). We attribute the large suppression at $\phi = 90°$ and $\phi = 270°$ to the anisotropy of weak (anti)localization. The other two lobes at $\phi = 0°$ and $\phi = 180°$ arise from the fourfold symmetric signal, which we attributed to magnetocrystalline AMR. This indicates that films of different thicknesses also develop a field-induced magnetization at low temperatures. The AMR appears to become larger as the film thickness is reduced, however its precise evolution should be subject to further investigation.
